# Wound healing potential of *Cystoseira*/mesenchymal stem cells in immunosuppressed rats supported by overwhelming immuno-inflammatory crosstalk

**DOI:** 10.1371/journal.pone.0300543

**Published:** 2024-04-04

**Authors:** Eman Maher Zahran, Reham H. Mohyeldin, Fatma Mohamed Abd El-Mordy, Sherif A. Maher, Nehad M. Reda Abdel-Maqsoud, Faisal H. Altemani, Naseh A. Algehainy, Mohammed A. Alanazi, Mohammed M. Jalal, Mahmoud A. Elrehany, Gerhard Bringmann, Usama Ramadan Abdelmohsen

**Affiliations:** 1 Faculty of Pharmacy, Department of Pharmacognosy, Deraya University, Minia, Egypt; 2 Faculty of Pharmacy, Department of Pharmacology, Deraya University, Minia, Egypt; 3 Faculty of Pharmacy (Girls), Department of Pharmacognosy and Medicinal Plants, Al-Azhar University, Cairo, Egypt; 4 Faculty of Pharmacy, Department of Biochemistry, New Valley University, New Valley, Egypt; 5 Faculty of Pharmacy, Department of Pathology, Deraya University, Minia, Egypt; 6 Faculty of Applied Medical Sciences, Department of Medical Laboratory Technology, University of Tabuk, Tabuk, Saudi Arabia; 7 Faculty of Pharmacy, Department of Biochemistry, Deraya University, Minia, Egypt; 8 Institute of Organic Chemistry, University of Würzburg, Am Hubland, Würzburg, Germany; 9 Faculty of Pharmacy, Department of Pharmacognosy, Minia University, Minia, Egypt; Helwan University, EGYPT

## Abstract

Wound healing, one of the most intricate and dynamic processes of the body, maintains skin integrity following trauma. One of the main issues that still exists is impaired wound healing, particularly for immunosuppressed patients. Recently, natural products from marine environments have been employed in wound-repairing activities. This work investigates the mesenchymal stem cells in the combined capacity of the bone marrow (BMMSC) for wound healing and *Cystoseira* sp. Algae extract in immunosuppressed rats. High-resolution liquid chromatography / MS investigation of *Cystoseira* extract revealed the prevalence of fatty acids that have wound-soothing potential. From constructed PPI network for wound healing and further analysis through molecular docking and molecular dynamics (MD) simulation experiments suggested that cystalgerone metabolite may be responsible for the wound healing-promoting effect of *Cystoseira* extract. According to the CD marker characterization of the BMMSC, 98.21% of them expressed CD90, and 97.1% expressed CD105. Sixteen d after immunity suppression (by 40 mg/kg hydrocortisone daily), an incision was made in the dorsal skin of the rat. The treatments were applied for 16 d and samples were taken from the tested groups on the 8^th^, 14^th^, and 16^th^ days. The BMMSCs / *Cystoseira* group showed significantly improved wound closure, thickness, density of new layers, and skin elasticity than the control group (p < 0.001). The BMMSCs / *Cystoseira* combination significantly reduced the oxidative indicators, pro-inflammatory cytokines, and immune markers, according to the RT-PCR gene expression study. In order to delve deeper into the complex interconnections among wound healing-related biological targets and pinpoint key factors in this complex process, we engaged in network pharmacology and computational research. Subsequently, we conducted a comprehensive computational analysis, including reverse docking, free energy (ΔG) computation, and molecular dynamics simulations, on the molecular structures of the annotated compounds. The purpose of this investigation was to identify potential new targets for these chemicals as well as any potential interactions they may have with different signaling pathways related to the wound healing process. Our research indicates that the primary compounds of *Cystoseira* holds potential wound healing therapeutic activity. Although more safety testing and clinical studies are required, the combination has great potential for regenerative medicine and could be a revolutionary advance in the healing of the wounds of immunosuppressed patients.

## 1. Introduction

A wound is a disruption of the cellular, functional, and anatomical integrity of living tissue, resulting from physical, chemical, or microbial threats [1]. Wound healing is a complex process occurring by regeneration or reconstruction of damaged tissue, which is essential for maintaining the protective / defense mechanisms of the skin [2]. In everyday practice and clinics, wound healing problems and their consequences provide a significant medical and multidisciplinary issue, particularly in immunocompromised individuals who heal slowly [3].

Tissue regeneration is regulated by the immune system, and signals from damaged cells drive circulating leucocytes to the site of injury. Many pro-inflammatory cytokines, including IL-1β, IL-6, TNF-α, and chemokines, are released by innate immune cells as soon as an injury occurs, which initiate the acute phase of the inflammatory response. Recent research has demonstrated that long-term exposure to high glucocorticoid levels might cause the body to become immunosuppressed and wound healing would be greatly affected [4]. This may be explained by the excessive production of reactive oxygen species (ROS), impaired ROS detoxification, and an altered inflammatory profile, which results in oxidative damage, the primary reason for delayed healing [5].

Mesenchymal stem cells possess the ability to differentiate into T cells and have traits related to immunomodulation [6]. They play a central role in tissue repair, in addition to having anti-fibrotic, anti-tumorigenic, anti-apoptotic, pro-angiogenic, neuroprotective, anti-inflammatory, anti-bacterial, and chemo-attractive properties [6]. In the past, BMMSCs were employed in the therapy of autoimmune disorders, graft-vs.-host disease (GVHD) [7], and other illnesses involving tissue regeneration and repair [8]. Moreover, the therapy of cancer, diabetes, cirrhosis, multiple sclerosis, myocardial infarction, and stroke is now within the clinical potential of BMMSCs [9]. Stem cells have been extracted from various tissues, such as adipose tissue, fetal tissues, dental pulps, and the umbilical cord but most preclinical investigations have used bone marrow-derived mesenchymal stem cells (BMMSCs) [10,11]. It has recently been shown that BMMSCs can regulate immunological responses, participating in both innate and adaptive immunity by interacting with monocytes and T-regulatory cells [12]. Adult stem cells (ASCs) have the ability to differentiate into specific cell types within a tissue or organ, while embryonic stem cells have the capacity to develop into all adult body cell types. Adult stem cells (ASCs) are acknowledged as key players in tissue regeneration since they are thought to be a source of replenishing cells lost during wound healing [13].

Bone marrow has two types of cells CD34^+^ and CD34^−^. Of these, CD34^−^ cells can differentiate into a wide variety of cell types, whereas CD34^+^ cells create blood cells [14]. Although bone marrow-derived BMMSCs are generally rare in the bone marrow, they are clonable and can be created up to a million times in vitro, yielding substantial quantities of BMMSCs for cell therapy. As a result, BMMSCs have already been employed in preliminary safety studies and clinical trials for the treatment of numerous illnesses [15]. On the other hand, natural products have, since ancient times, been utilized in many experimental models for therapeutic purposes [16,17]. The abundance of secondary metabolites found in the marine environment has long been used, particularly in the case of seaweeds, which are used as a food source in many parts of the world [18]. They are reported to contain an efficient antioxidative defense system, as a result of exposure to adverse environmental conditions, such as solar light, pH disturbance, and oxidative stress [19].

During the past years, interest in marine species as a hopeful source of pharmacologically active compounds has grown, owing to their capacity to produce a wide range of bioactive compounds of interest for medical applications [20]. The genus *Cystoseira* (Sargassaceae) contains roughly 40 species of marine brown algae, which is frequently found along the eastern Atlantic and Mediterranean coasts [21]. The biological significance of genus *Cystoseira* stems from the presence of terpenoids, steroids, phenolic compounds, phlorotannins, triacylglycerols / fatty acids, pigments, and vitamins [22]. Some of the isolated compounds had various pharmacological effects, including antioxidant, anti-inflammatory, cytotoxicity, anticancer, cholinesterase inhibition, anti-diabetic, or antimicrobial activities [23]. In recent years, stem cell treatment has emerged as a highly promising and advanced scientific field of study [24]. Despite this, previous research indicates that an extract from brown algae (*Cystoseira myrica*) can significantly accelerate wound healing in a wound model with full-thickness skin [25]. However, no research has been done on the possibility of *Cystoseira* algae for wound healing.

*Cytoseira* species were reported to contain lipids, particularly fatty acids, which have been shown in numerous studies to be useful in controlling wound healing [26]. Since they have anti-inflammatory qualities and can stimulate tissue repair and cell regeneration, fatty acids are essential for wound healing [27]. It has been demonstrated that they lessen inflammation, stop the growth of bacteria, and increase collagen synthesis, all of which are necessary for the closure of wounds. Furthermore, fatty acids assist in keeping the wound bed properly hydrated and wet, avoiding excessive drying or exudate generation [28]. It has also been discovered that fatty acids improve the immune response by stimulating specific immune cells and encouraging a well-balanced inflammatory response. Fatty acids support tissue regeneration, collagen formation, inflammation control, and immune modulation, among other aspects of wound healing [27,28].

**Fig 1:**
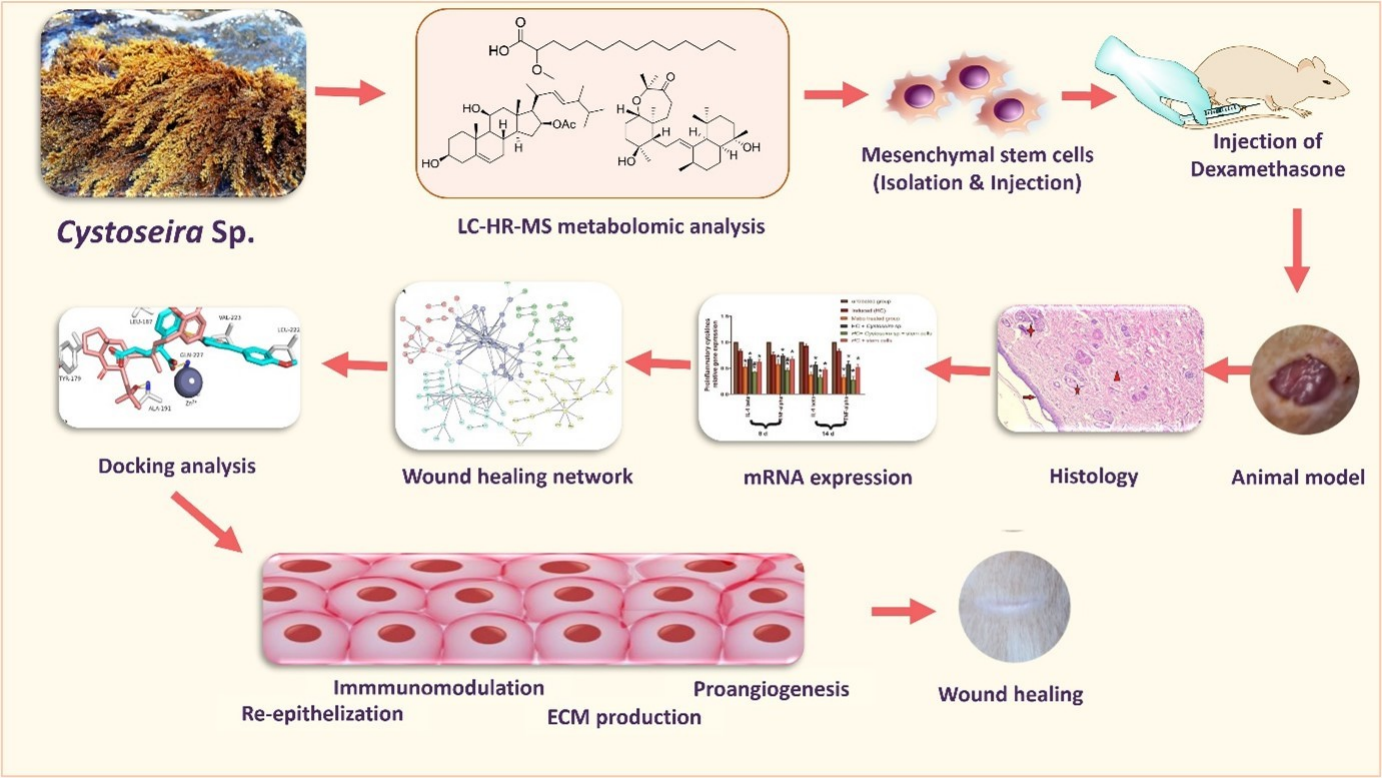
Workflow of the study.

The current study aimed to assess the wound-healing efficacy of *Cystoseira* brown algae extract alone and in combination with stem cell therapy. Additionally, we aimed to investigate the metabolomic profiling secondary metabolites of the brown alga *Cystoseira* using LC-HR-ESI-MS and evaluate its potential for wound healing in immunocompromised mice in conjunction with BMMSC injection, monitored by histopathological as well as PCR analytical techniques (Fig 1).

## 2. Methods

The necessary institutional, national, and international guidelines were adhered to during the conduct of all experiments.

### 2.1. Metabolomic analysis of *Cystoseira sp*. extract

#### 2.1.1. Gathering and identifying *Cystoseira* samples

*Cystoseira* specimens were gathered in May 2022 from the Red Sea coast of Safaga City, Red Sea Governorate, Egypt. The collected samples were cleaned using tap water, distilled water, and seawater in that order. The materials were transported to the lab in an iced box after being stored in sterile plastic vials and kept cold. A voucher specimen (2023-Der12) was kept at the Pharmacognosy Department, Faculty of Pharmacy, Deraya University in Egypt.

#### 2.1.2. Extraction of *Cystoseira* sp

Samples of *Cystoseira sp*. weighing about 250 g were gathered, let to air dry in the shade for a month, and then processed into a fine powder using an OC-60B/60B grinder (60–120 mesh, Henan, Taiwan). The powder was macerated and extracted for 3 d each with 5 L of 70% ethanol. It was then concentrated under vacuum at 45°C using a rotary evaporator (Buchi Rotavapor R-300, Cole-Parmer, Vernon Hills, IL, USA) to yield 30 g of crude extract [29].

#### 2.1.3. Metabolic analysis

A Synapt G2 HDMS quadrupole hybrid mass spectrometer (Waters, Milford, CT, USA) was used to perform metabolic tentative identification by high-resolution liquid chromatography-mass (HR-LC-MS), and the Dictionary of Natural Products (DNP) database was used to assist with the tentative identification of the compounds [30].

### 2.2. Biological study

#### 2.2.1. The excision wound model in rats

The experiment entailed habituating 36 male albino rats, who were in a state of optimal physical condition, with an average weight of 150 grams. The rats were randomly divided into six groups, each including six animals. The excision model was carried out following the ARRIVE criteria, with approval number 10/2022 dated July 26, 2022, from the Ethical Committee at Deraya University [31].

- **Wound creation**:

An intraperitoneal injection of a ketamine-xylazine combination was used to anesthetize the animals. Prior to the formation of the wound, a combination of xylazine (10 mg/kg body weight) and ketamine (90 mg/kg body weight) was administered. A fur clipper was used to shave the dorsal surface of the rats, followed by spraying 70% ethanol to ensure thorough skin cleansing. With the use of a sterile surgical blade and scissors, a completely sterile excision wound measuring 88 mm was created. Subsequently, an application of a topical pain reliever (buprenorphine, 0.5 mg/kg body weight) was made. With a focus on minimizing pain and alleviating suffering, every effort was made to decrease pain tolerance [32,33].

Six sets of six rats each that were randomly assigned; the excision model was conducted in the following manner:**Group I: (Negative control);** The vehicle of the plant extract was applied topically to the rats twice daily for a period of sixteen days following the wounding, and the rats were also given an intramuscular injection of the HC vehicle to administer 7 days before the wounding.

**Group II: (Immunosuppressed rats);** rats received intramuscular injection of HC at a dose of 40 mg/kg per day, for 7 days prior to wounding, + application of the vehicle of the plant extract (CMC) should be performed twice daily for a period of 16 days following the development of the wound [34].

**Group III: (Standard group);** In this experimental group, the rats were administered a daily dose of 40 mg/kg of HC for 7 days before the wound was created. Additionally, they received topical application of Mebo^®^ twice daily for 16 d following the development of the wound, as a standard preparation [34].

**Group IV: (BMMSCS treated group);** rats received intramuscular injection of HC at a dose of 40 mg/kg per day, for 7 d prior to wounding [34] + BMMSCs (1×10^6^ cells) were intravenously administered *via* the lateral tail vein of the rat following 24 h of wound creation.

**Group V: (Plant extract treated group);** rats received intramuscular injection of HC at a dose of 40 mg/kg per day, for 7 d prior to wounding [34] + topical application of plant extract twice daily for 16 d after wound creation.

**Group VI: (Combination group);** rats received intramuscular injection of HC at a dose of 40 mg/kg per day, for 7 d prior to wounding [34] +BMMSCs (1×10^6^cell) were intravenously (i.v.) administered *via* the lateral tail vein of the rat following 24 h of wound creation + topical application of the plant extract twice daily for 16 d after wound creation.

- **Collection of tissue samples, calculation of percentage wound closure rate**

Full-thickness skin biopsies of the complete ulcers from each tested group were taken on days eight and 14 while the patient was under anesthesia. The tissue samples were divided into three sections for gene expression analysis.

On day 16, skin samples were collected from each group and preserved in paraffin. The sections were then stained with hematoxylin and eosin as well as trichrome staining methods for histological analysis.

A camera was utilized to monitor the progress of the wound area at 3-day intervals until complete healing was achieved. Image J 1.49v software was utilized to evaluate the wound area. The rate of wound closure was determined using a formula that calculates the percentage change in the initial wound area.

$$wound\;closure\;\left(\text{\%}\right)=\frac{\left(area\;of\;wound\;on\;day\;0-area\;of\;wound\;on\;day\;nth\right)}{\left(area\;of\;wound\;on\;day\;0\right)}\times 100,$$

where n represents the order of the examination day, i.e., the 4th, 6th, 8th, 14^th^, and 16^th^ day.

#### 2.2.2. Mesenchymal stem cells: Isolation, culture, characterization, and injection

- **BMMSC isolation from bone marrow**

As previously reported by Zahran *et al*., 2023 [35], the mesenchymal bone marrow stem cells were acquired from Nawah Centre in Cairo, Egypt, which separated and characterized the cells. After a BMMSC culture and trypan blue exclusion test to determine cell viability, BMMSCs were characterized by flow cytometric phenotyping analysis. The BMMSCs were eventually injected in accordance with Zahran *et al*., 2023 [35] and the supporting data.

#### 2.2.3. Histopathological study

Samples of dorsal skin were collected, allowed to settle in buffered formalin, and then processed *via* a grading system of xylene and alcohol before being placed into paraffin blocks. Tissue strips of 5–6 μm in thickness were stained with hematoxylin/eosin and a specific Masson’s trichrome in order to determine the density of collagen fibers. The Leica Application Suite (Leica Microsystems, Wetzlar, a light microscope) was used to examine and take pictures of the prepared slides [4].

#### 2.2.4. Gene expression analysis

By using the TRIzol reagent (Invitrogen, USA), in accordance with the manufacturer’s instructions, total ribonucleic acid (RNA) was extracted from skin tissues. Using a Nano Drop 1000 (Thermo Scientific, Waltham, MA, USA), the amount of extracted RNA was measured spectrophotometrically. Using oligo-dT primers and a high-capacity reverse transcription kit (Thermo Scientific, USA), complementary deoxyribonucleic acid (cDNA) was reverse-transcribed from 1 μg total RNA. Real-time polymerase chain reaction (PCR) was used to determine transcript levels using sequence-specific primers provided in S1 Table. The SYBR Green PCR Master Mix (Thermo Scientific, USA) was controlled by the manufacturer using a step one real-time PCR thermal cycler (Thermo Fisher, USA) for amplification. The comparative CT approach was used to evaluate the gene expression levels following normalization to GAPDH as a housekeeping gene [4].

#### 2.2.5. Statistical analysis

A two-way ANOVA test and a one-way ANOVA followed by Bonferroni’s multiple comparisons tests were performed to assess if there was a significant difference between the groups. The mean ± SD is used to present the data. * p < 0.05 compared to the untreated group on the same day, and # p < 0.05 compared to the Mebo^®^ group on the same day. (B) To clarify observed differences in the shape and direction of wound contraction between groups, the wound aspect ratio was calculated (length: width).

### 2.3. Network pharmacology

#### 2.3.1. Bioinformatics-based investigation of protein-protein interactions in wound healing process

This study entails a nuanced exploration of the interplay among proteins implicated in the wound-healing process. Initially, it entailed an extensive assessment of human proteins associated with the wound-healing process. To compile this data, a methodical search was executed, employing “wound healing” and "human" as the key search terms. This search encompassed an examination of the Toxicogenomics database (https://ctdbase.org/) and GeneCards, along with a detailed review of existing scholarly literature. This meticulous approach yielded the identification of a substantial corpus of over 224 proteins tied to wound wound-healing process as detailed in S2 Table. To advance this analysis, the study utilized the capabilities of Cytoscape software, leveraging it to meticulously construct a network model of protein-protein interactions (PPI), centered on these 224 specifically identified proteins. This approach enabled a more structured and insightful exploration of the complex protein interactions inherent in the wound-healing process.

#### 2.3.2. Predicting target proteins for the LC-MS annotated compounds

A series of computer-based assessments were conducted on the LC-MS annotated compounds to assess their potential as wound-healing-promoting agents. The digital models of these compounds were analyzed using the PharmMapper platform (http://www.lilab-ecust.cn/pharmmapper/) to investigate their ability to bind with cancer-related protein targets. PharmMapper, a novel platform employing pharmacophore-based virtual screening, aligns a given structure with the pharmacophore maps derived from the 3D active sites of proteins listed in the Protein Data Bank (PDB; https://www.rcsb.org/) [36].

#### 2.3.3. Docking and molecular dynamics simulation

Docking and MD simulation was carried out according to our previously reported method and the full procedures were described in detail in the supplementary file.

## 3. Results

### 3.1. Metabolomic profiling of *Cystoseira* algae

**Fig 2:**
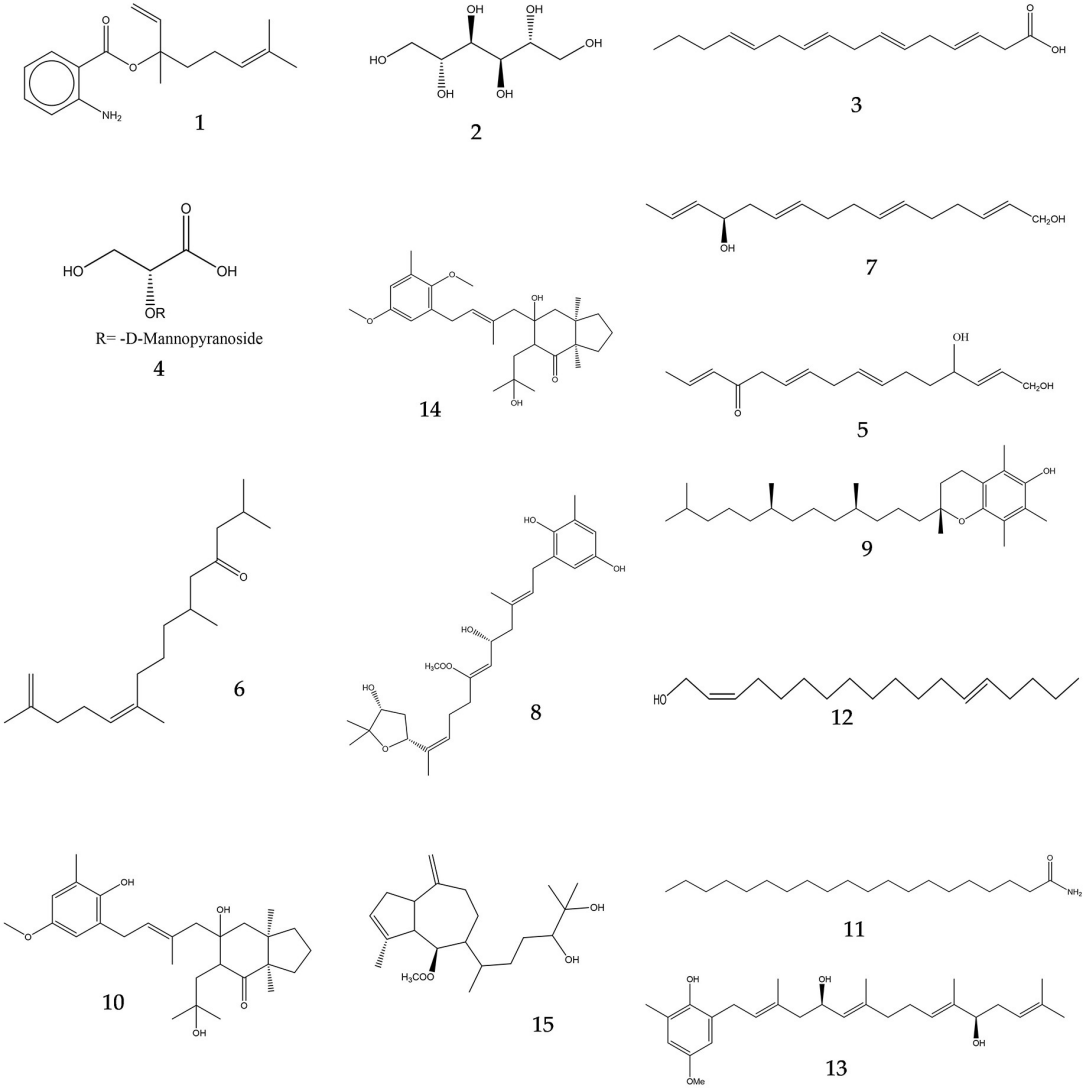
Structures of dereplicated compounds from *Cystoseira* algae.

Metabolic profiling was carried out using LC-HR-ESI-MS to tentatively identify active constituents that are responsible for the wound healing potential of *Cystoseira* algae. LC-HR-ESI-MS dereplication led to the identification of several metabolites belonging to various chemical classes, of which fatty acids were predominant. Identification of these metabolites was performed by comparison with data reported in the literature, by which 15 compounds were identified (S3 Table, Fig 2).

Fatty acids metabolites are thought to encourage wound healing as reported [37]. From the dereplicated data, the mass ion peak at *m/z* 274.1727 [M + H]^+^, in accordance with the molecular formula C_17_H_23_NO_2_, was identified as 1,6-octadien-3-ol, 3,7-dimethyl-, 2-aminobenzoate, which had formerly been obtained from *Cystoseira crinite* [23]. The mass ion peak at *m/z* 183.0791 [M + H]^+^ for the suggested molecular formulas C_6_H_14_O_6_ was recognized as mannitol, previously identified in *Cystoseira nodicaulisi* [23]. Additionally, another metabolite (C_16_H_26_O_2_), with a mass ion peak at *m/z* 251.1931 [M + H]^+^, was identified as hexadeca-4,7,10,13-tetraenoic acid, as formerly reported in *Cystoseira crinite* [23]. The metabolite with the molecular formula C_9_H_16_O_9_, known as 2,3-dihydroxypropanoic acid, as its (*R*)-enantiomer, 2-O-α-D-mannopyranoside, was dereplicated from the mass ion peak at *m/z* 269.0795 [M + H]^+^, as previously isolated from red algae *Alsidium*, *Chondria*, *Laurencia*, *Polysiphonia*, *Halopytis*, *Vidalia*, and *Digenea* spp. [38]. Moreover, the mass ion peak at *m/z* 319.2190 [M + H]^+^ for the predicted molecular formula C_20_H_30_O_3_ was distinguished as 1,4-dihydroxy-2,7(19),10,14-phytatetraen-13-one, as its (2E,4R,10E)-isomer, 4-ketone, which had previously been isolated from *Cystoseira crinite* [39]. The mass ion peak at *m/z* 281.2401 [M + H]^+^, for the suggested molecular formula C_18_H_32_O_2_, was identified as 6,10,14-trimethyl-5-pentadecene-2,12-dione, as its (E)-diastereomer, which had also been previously reported as a constituent of the brown alga *Sargassum micracanthum* [40]. Furthermore, the mass ion peak at *m/z* 305.2404 [M + H]^+^, for the predicted molecular formula C_20_H_32_O_2_, was identified as 2,6,10,14-phytatetraene-1,13-diol, as its (2E,6E,10E,13R)-form, 13-ketone, which had earlier been reported from *Cystoseira elegans* and *Cystoseira balearica* [41]. The mass ion peak at *m/z* 485.2826 [M + H]^+^, in accordance with the molecular formula C_29_H_40_O_6_ was distinguished as zosterdiol A; 1’,4’-Di-de-Me, 1’,4’-quinone, 5-acetyl, which had previously been isolated from *Cystoseira crinite* [42]. The metabolite α-tocopherol (C_29_H_50_O_2_) was dereplicated from the mass ion peak at *m/z* 431.3813 [M + H]^+^, which had been previously obtained from *Cystoseira barbata* [23]. Additionally, the metabolite cystalgerone, O1’-demethyl, was dereplicated from its mass ion peak at *m/z* 441.2928 [M + H]^+^, in accordance with the molecular formula C_28_H_40_O_4_; it had previously been isolated from *Cystoseira algeriensis*, *Cystoseira marina*, and *Cystoseira baccata* [43]. Besides, the one with a molecular formula of C_20_H_41_NO, and with a mass ion peak at *m/z* 312.3186 [M + H]^+^, was detected as eicosanoic acid amide [44]. 2,13-Octadecadien-1-ol, with the formula C_18_H_34_O, was dereplicated from the mass ion peak at *m/z* 267.2609 [M + H]^+^ [45]. Moreover, the mass ion peak at *m/z* 441.2927 [M + H]^+^, for the predicted molecular formula C_28_H_40_O_4_ was distinguished as 1-(2-hydroxy-5-methoxy-3-methylphenyl)-3,7,11,15-tetramethyl-2,6,10,14-hexadecatetraene-5,12-diol as its 2E,5R,6E,10E,12R-isomer, 12-ketone, which had formerly been reported as a constituent in *Cystoseira* sp. [41]. The mass ion peak at *m/z* 455.3085 [M + H]^+^, in accordance with the molecular formula C_29_H_42_O_4_, [M + H]^+^, in accordance with the molecular formula C_29_H_42_O_4_, was recognized as cystalgerone, which had previously been identified as a metabolite from *Cystoseira algeriensis* and *Cystoseira platyramosa* [46]. The metabolite 3,10(18)-pachydictyadiene-6,14,15-triol, in its (1α,5β,6β,11R,14S)-form, 6-acetyl, with the molecular formula C_22_H_36_O_4_, was also dereplicated from the mass ion peak at *m/z* 365.2615 [M + H]^+^; this compound had earlier been isolated from *Cystoseira myrica* [47].

### 3.2. Biological study

#### 3.2.1. Macroscopical assessment and estimation of the rates wound closure

**Fig 3:**
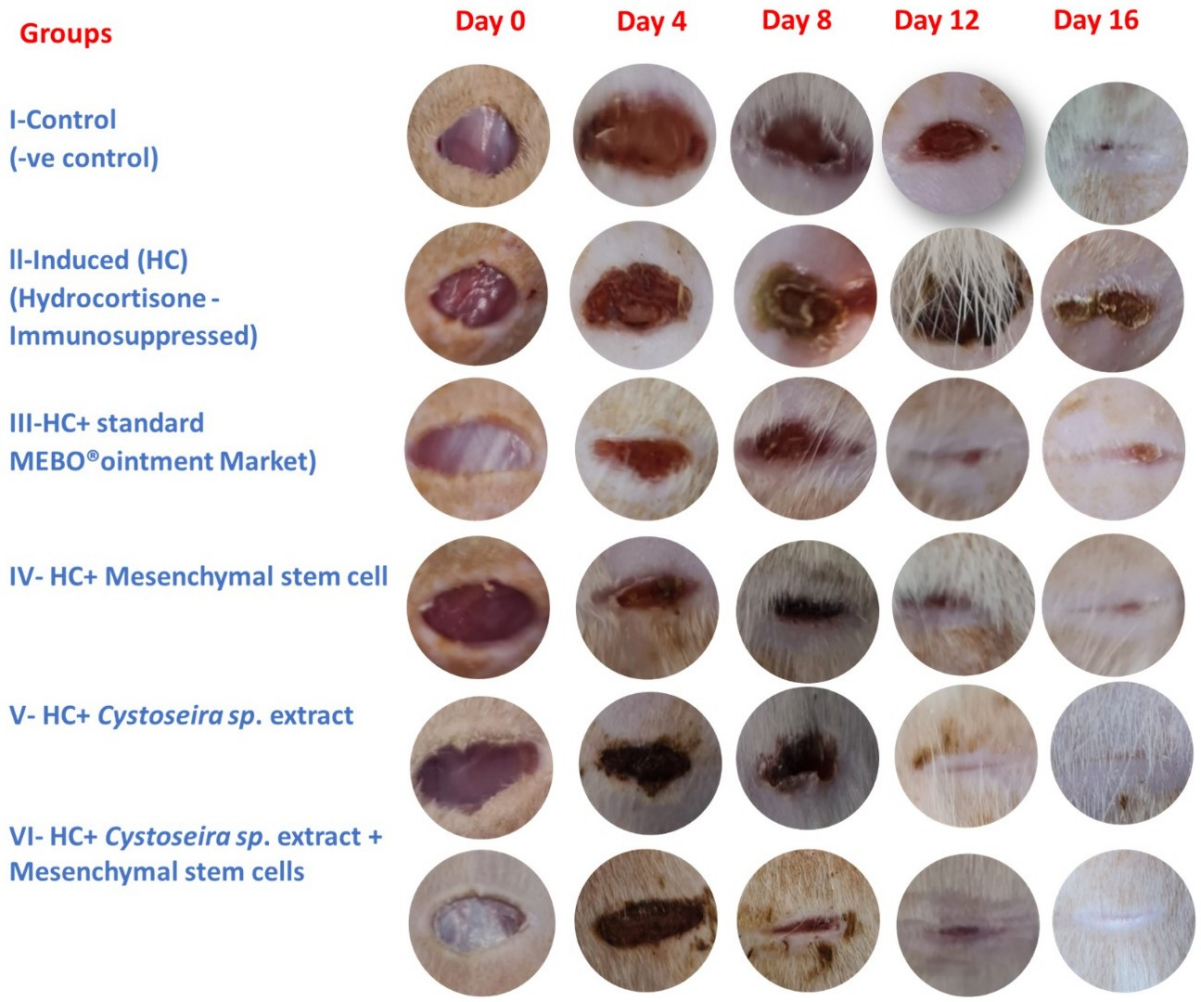
Mesenchymal stem cell infusion on skin wound healing of hydrocortisone-immunosuppressed Wistar rats. Progress of skin wound healing over 16 days of observation. The table columns show the experimental groups, with the control group **(I),** hydrocortisone group (**II**), Mebo^®^ ointment Market group (**III**), hydrocortisone-treated with mesenchymal stem cells group (**IV**), hydrocortisone-treated with *Cystoseira sp*. extract group (**V**) and hydrocortisone-treated with *Cystoseira sp*. extract + mesenchymal stem cell group (**VI**).

**Fig 4:**
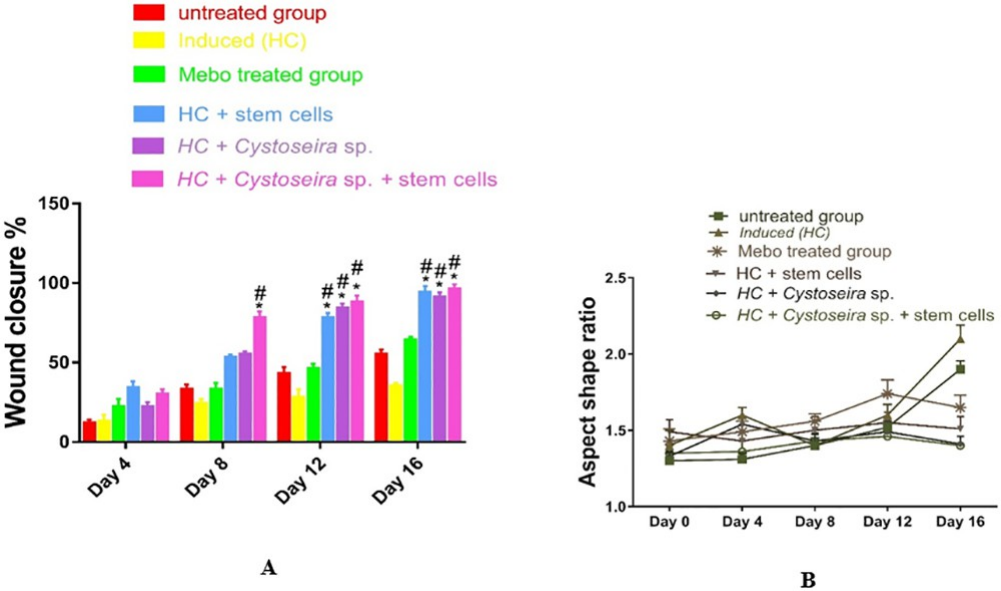
**(A)** Wound closure rates over time post-injury in all experimental groups, (**B**) the wound aspect ratio was calculated to describe observed changes in the shape and direction of wound contraction between the groups (length: width).

The macroscopical observations made on days 0, 4, 8, 12, and 16 showed that the wound closure rate of the treated groups had significantly increased, with the best results obtained in group VI, nevertheless, by day 16, the wound had almost completely healed, as seen in Figs 3 and 4 illustrates the semi-quantitative wound closure rate of the six groups (n = 6), where Fig 4A represents the percentage of wound closure, estimated by Image J with time after injury (0, 4, 8, 12, and 16 d). An indicator of wound closure is the centripetal flow of the edges of a full-thickness wound. However, group 6 is shown in Fig 4B as having the lowest aspect shape ratio, which reflects the variations in wound contraction direction and shape that have been noted between the groups. Group 6 has the greatest rate of wound repair and the lowest aspect shape ratio, indicating that both the extract and the BMMSCs had improved effects, leading to virtually normal skin appearance again.

#### 3.2.2. BMMSC immunophenotyping

**Fig 5:**
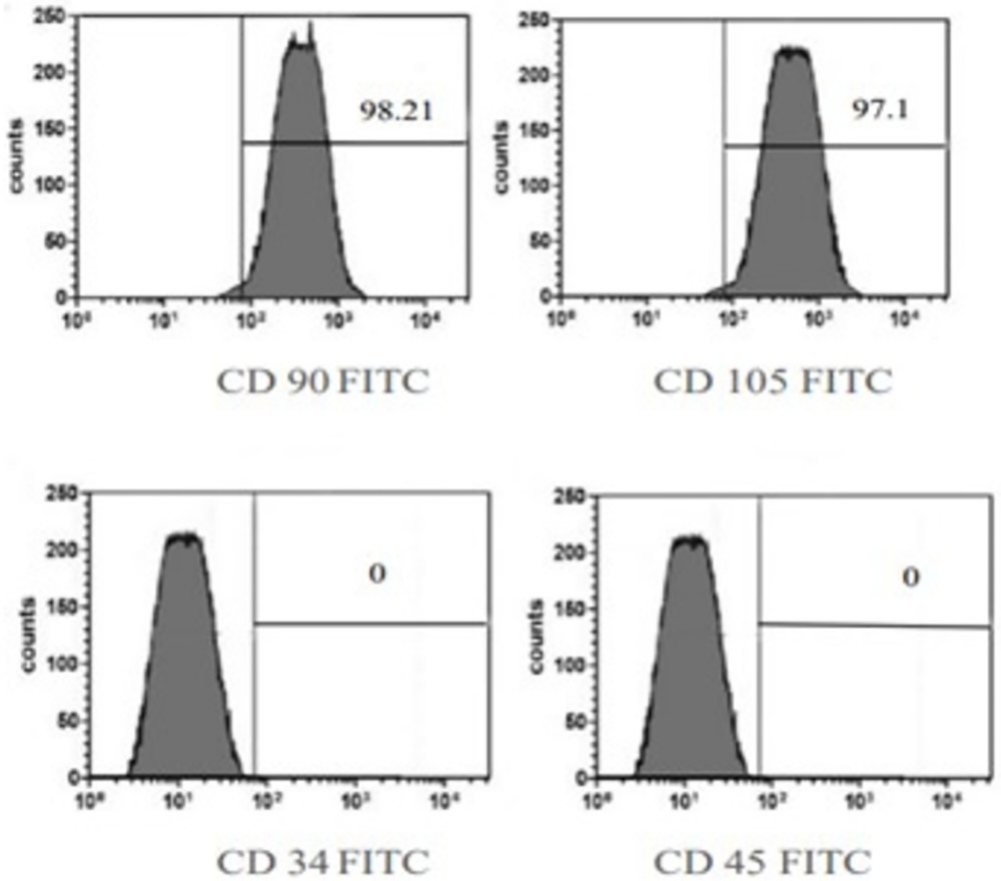
Flow cytometric analysis showed that cultured immunophenotype of BMMSC displayed negative expression of CD34 and CD 45, also highlighting positive expression of CD90 (98.21%) and CD105 (97.1%) antibodies staining.

Following the guidelines of the Mesenchymal and Tissue Stem Cell Committee of the International Society for Cellular Therapy, a flowcytometric analysis of the BMMSC surface markers was carried out following bone marrow isolation to verify the identity of the cells as BMMSCs and not hematopoietic stem cells [48]. The information showed that mesenchymal markers, such as CD90 and CD105 were positively expressed by 98.21% and 97.1% respectively, while CD34 and CD45 markers were negatively expressed (Fig 5).

#### 3.2.3. Results of histopathology

**Fig 6:**
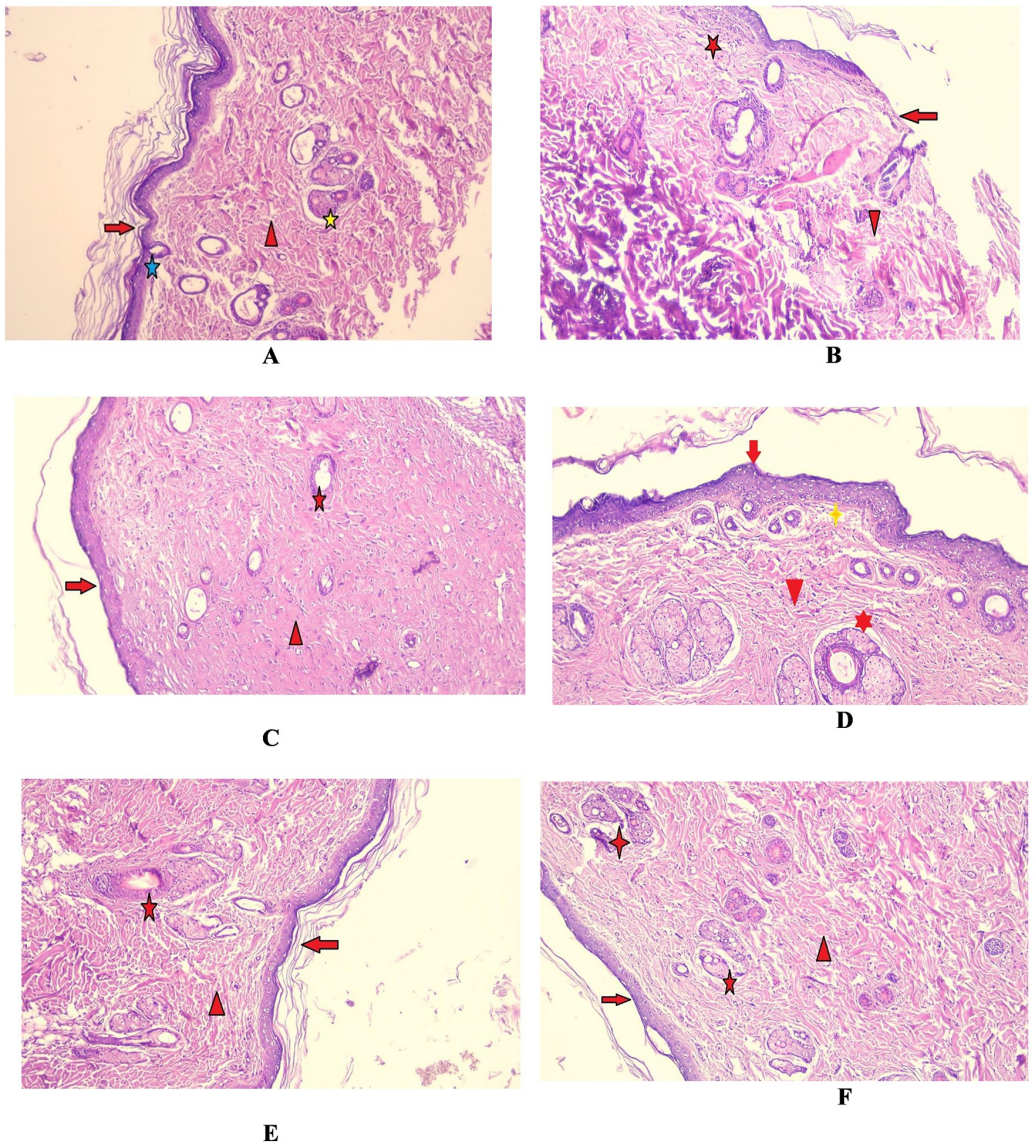
**(A)** (**Group I**) showing the mature epidermis with keratinization (red arrow), subepidermal and upper dermal layers with dermal collagen (red arrowhead), skin adnexa (yellow star), and hair follicle (blue star), 200x; (**B**) (**Group II**) showing discontinuous epidermal skin layers covered with necrotic tissue slough and blood clots (red arrow), dermal layers with mild edema (red arrowhead) and inflammatory cellular infiltrated macrophages (red star), 200x; **(C) (Group III)** showing epidermal skin layer with thin keratinization (red arrow), dermal layer with thick wavy compactly organized collagen bundles (red arrowhead) and folliculosebaceous unit of skin adnexa (red star). 200x; (**D**) (**Group IV**) section showing normal mature epidermal skin layer with keratinization (red arrow). Dermal layer shows compactly organized collagen bundles (red arrowhead) with mature folliculosebaceous unit of skin adnexa (red star). Mild subepidermal edema was also noticed (yellow star) 200x; (**E**) (**Group V**) section displayed a normal mature epidermal skin layer with keratinization (red arrow). Dermal layer showing mature organized wavy collagen bundles (red arrowhead) with scattered mature folliculosebaceous unit of skin adnexa (red star), 200x; (**F**) (**Group VI**) showing normal mature epidermal skin layer with keratinization (red arrow), dermal layer showing compactly organized mature thick collagen bundles (red arrowhead) with multiple mature folliculosebaceous of skin adnexa (red star), 200x.

The following histological findings from the sections under examination and the results of the gross evaluation were in agreement:

***Group I (negative control rats)*:** The skin sections displayed a mature continuous stratified squamous epidermal layer with keratinization, the subepidermal and upper dermal layers showing compactly organized dermal collagen, skin adnexa in form of mature sebaceous units and hair follicles, see Fig 6A.
***Group II (immunosuppressed rats without treatment)*:** The wound surface showed discontinuous epidermal skin layers covered with necrotic tissue slough and many blood clots. The dermal layers showed scars with mild edema and chronic inflammatory cellular infiltrates, where macrophages appeared in the healed areas for the organization (Fig 6B).
***Group III (immunosuppressed rats + Mebo***^***®***^***)*:** The normal mature epidermal skin layer appeared with thin keratinization. The dermis appeared to show thick wavy compactly organized collagen bundles with early developed folliculosebaceous unit of skin adnexa, congested blood capillaries, and blood clots (Fig 6C).
***Group IV (immunosuppressed rats + BMMSCs)*:** Section showing a normal mature epidermal skin layer with keratinization, while the dermal layer shows compactly organized collagen bundles with mature folliculosebaceous unit of skin adnexa with the presence of mild subepidermal edema (Fig 6D).
***Group V (immunosuppressed rats + Cystoseira extract)*:** The skin section showed a normal mature epidermal skin layer with keratinization. The dermal layer showed mature organized wavy collagen bundles with scattered mature folliculosebaceous units of skin adnexa (Fig 6E).
***Group VI (immunosuppressed rats + BMMSCs + Cystoseira extract)*:** The skin sections exhibited a mostly normal appearance, with thin scar tissue (keratinization) covering the typical stratified squamous keratinized epithelium. The dermal layer showed compactly organized mature thick collagen bundles (red arrowhead) with multiple mature folliculosebaceous units of skin adnexa, numerous blood capillaries, and hair follicles, with no inflammatory cellular infiltration (Fig 6F).

The net result revealed that the best results were obtained by combining the positive effects of both the extract and the BMMSCs, where the skin tissues appeared nearly normal.

#### 3.2.4. Results of gene expression

- ***Impact of BMMSCs and Cystoseira extract on expression of Cox-1*, *Cox-2*, *IL-1β*, *TNF-α*, *INF-ϒ*, *NF-KB*, *TGF-β*, *and IL-10***

**Fig 7:**
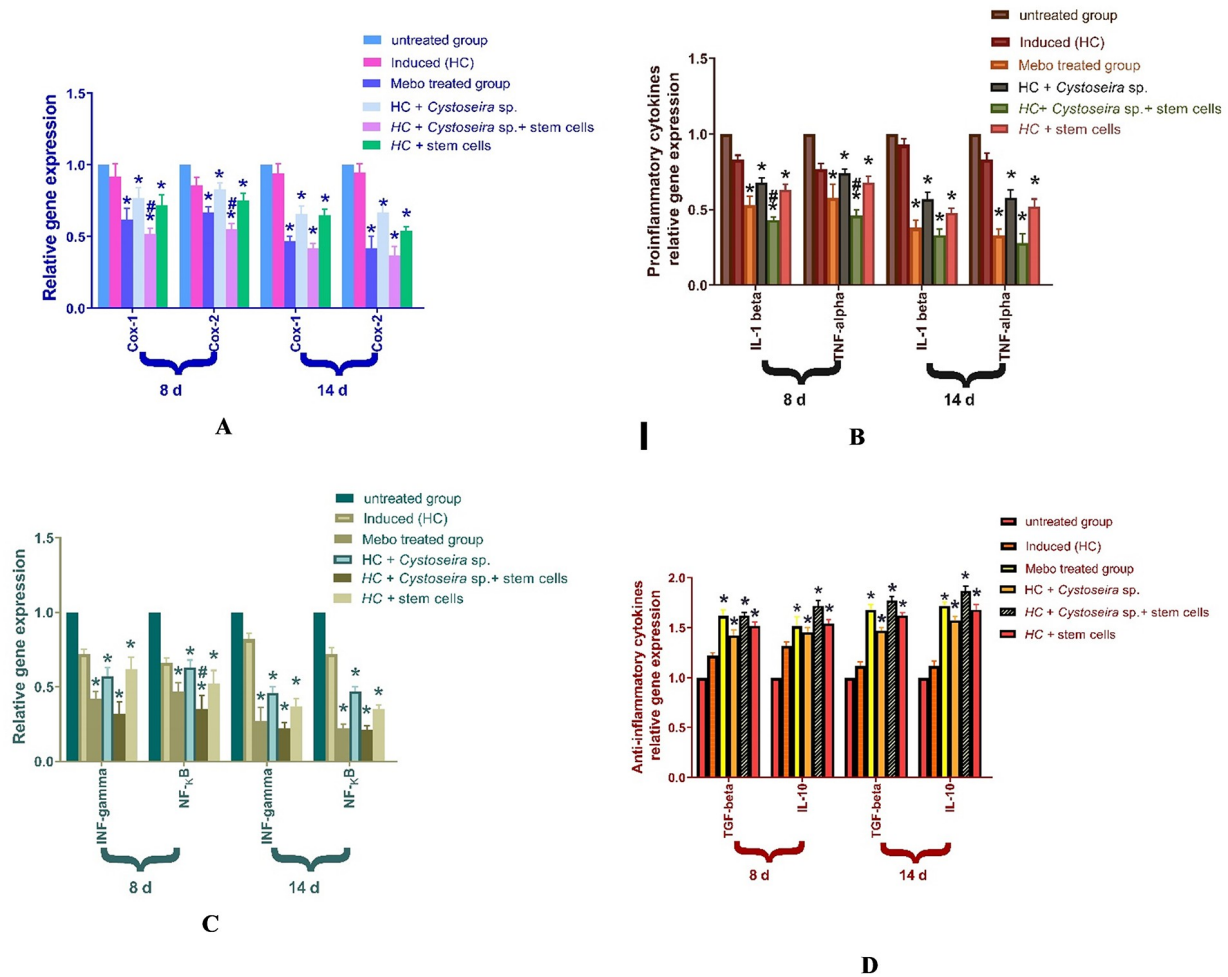
**(A)** mRNA expression of COX-I and COX-II; **(B)** mRNA expression of *IL-1β* and *TNF-α*; **(C)** mRNA expression of *INF-γ* and *NF-*_*K*_*B*; **(D)** mRNA expression of *TGF-β* and *IL-10* by quantitative RT-PCR. The data reflect fold change relative to the relative gene expression in the normal control group after being normalized to glyceraldehyde 3-phosphate dehydrogenase (*GAPDH*). The mean ± standard deviation is represented by bars and the significant difference among the groups is examined by a one-way ANOVA test, where: *p < 0.001 compared with those of the untreated group on the respective day and #p < 0.001 compared with those of the Mebo^®^ group on the corresponding day.

Fig 7A shows the mRNA expression of Cox-1 and of Cox-2 following an excisional wound treated with *Cystoseira* extract, BMMSCs, Mebo^®^, or *Cystoseira /* BMMSCs. The results revealed that, for 8 and 14 days, the skin tissues treated with *Cystoseira* and stem cells had significantly lower levels of Cox-1 and Cox-2 relative mRNA expression than the untreated *group* (p < 0.001). However, when compared to the Mebo^®^-treated group, the relative expression of the marker showed a substantial decrease in the *Cystoseira* / stem cells treated group.

As seen in Fig 7B, when comparing wounds treated with stem cells, *Cystoseira* extract, or Mebo^®^ to untreated wounds, the mRNA expression of *TNF-α* and *IL-1β* from full-thickness wound samples on day 8 post-injury showed a considerable downregulation of their activity. However, wounded rats treated with *Cystoseira* / stem cells displayed a considerably stronger reduction compared to the Mebo^®^-treated group. Moreover, stem cells and *Cystoseira* extract treatment or Mebo^®^ treatment for 14 d showed a remarkable reduction in *TNF-α* and *IL-1β* mRNA expression, compared to the untreated group at (*p* < 0.001), which was much more apparent in *Cystoseira /* stem cells-treated group.

On days 8 and 14 after injury, full-thickness wounds treated with stem cells, *Cystoseira* extract, or Mebo^®^ exhibited significantly lower levels of relative gene expression of INF-ϒ and NF-KB as compared to the untreated wounds, as Fig 7C illustrates. However, the *Cystoseira /* stem cells group displayed a significantly stronger downregulation compared to the Mebo^®^ group. The mRNA expression of the anti-inflammatory cytokines *TGF-β* and *IL-10* is shown in Fig 7D after stem cells, *Cystoseira*, and Mebo^®^ treatment. The *TGF-β and IL-10* relative mRNA expression in tissues was substantially elevated in stem cells and wounds treated with *Cystoseira* extract for 8 or 14 d compared to the untreated group (p < 0.001). Remarkably, the relative expression of the *Cystoseira* / stem cells-treated group, showed a significant elevation in the marker levels, in comparison with the Mebo^®^-treated group.

### 3.3. Network pharmacology

#### 3.3.1. Bioinformatics-based investigation of protein-protein interactions in wound healing process

**Fig 8:**
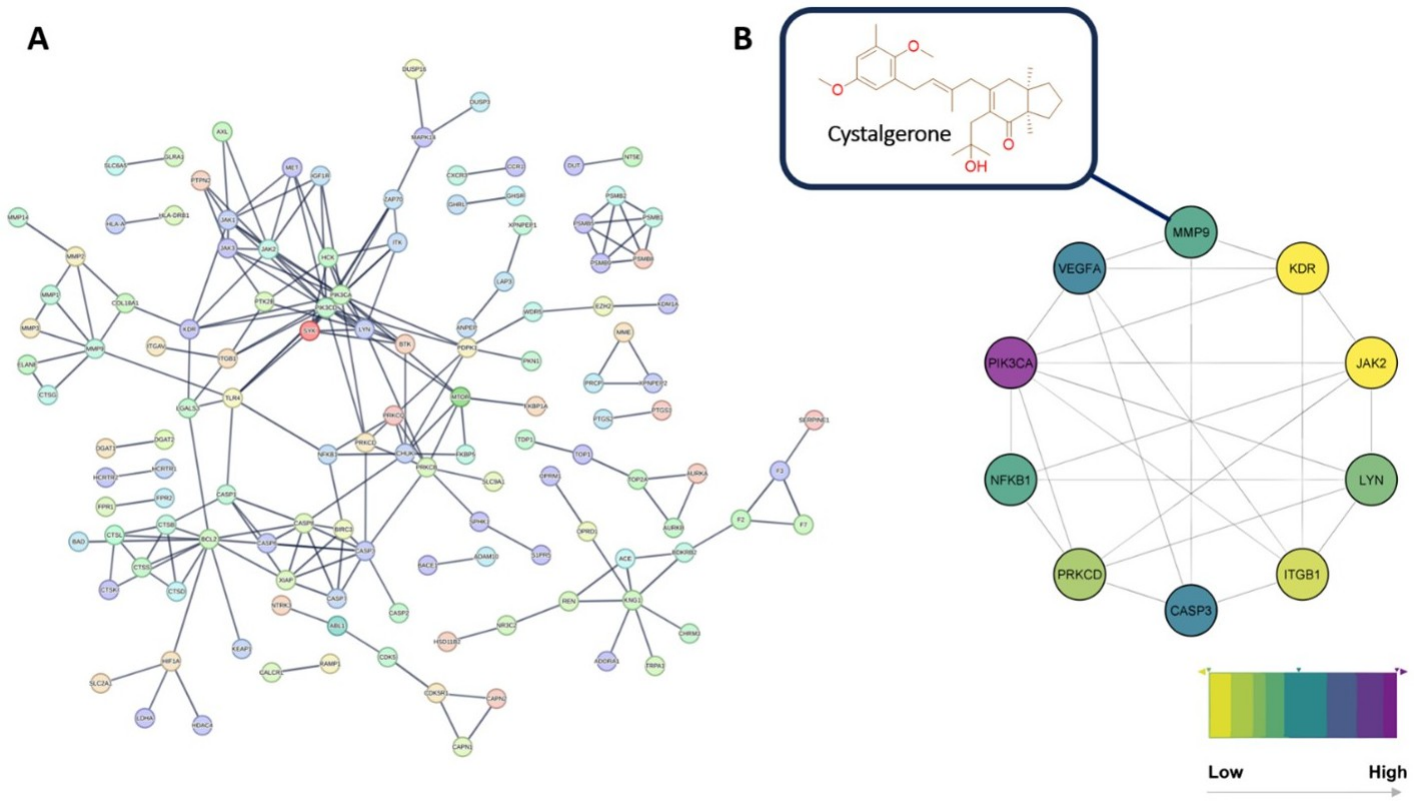
**(A)** Human cancer PPI network. This network consists of 201 nodes and 202 edges with an average node degree of 2.01. The top-interacting nodes were colored red (7.9%, 38 proteins of all interacting nodes, namely, hub protein). **(B)** The top interacting nodes (4.4%, ten proteins of all interacting nodes, namely, hub protein). Cystalgerone was predicted to interact with one of the hub proteins (viz., MMP9).

Fig 8 illustrates the intricate architecture of the developed protein-protein interaction (PPI) network, characterized by its intermediate connectivity. This is evidenced by the presence of 202 edges linking 201 nodes, an average node degree of 2.01, and a local clustering coefficient of 0.434. However, it was observed that a subset of proteins, specifically the remaining proteins in the list of 224 (S2 Table), did not exhibit any linkages within this network. Consequently, these non-connected proteins were excluded from the PPI network analysis.

The pursuit of wound healing therapies could be significantly enhanced by prioritizing proteins that demonstrate high levels of interaction within these networks. Such proteins, often referred to as hub proteins or genes, are typically central to the network and are deemed crucial molecular targets. This concept is supported by Meng *et al*. (2022) [49], who emphasize the significance of these hub proteins in therapeutic strategies. In line with this perspective, our study concentrated on the upper 4% of these proteins, amounting to ten proteins (Fig 8B). These were identified as the most highly interactive molecular targets within the network, selected based on their degree value, as depicted in Fig 8B.

**Fig 9:**
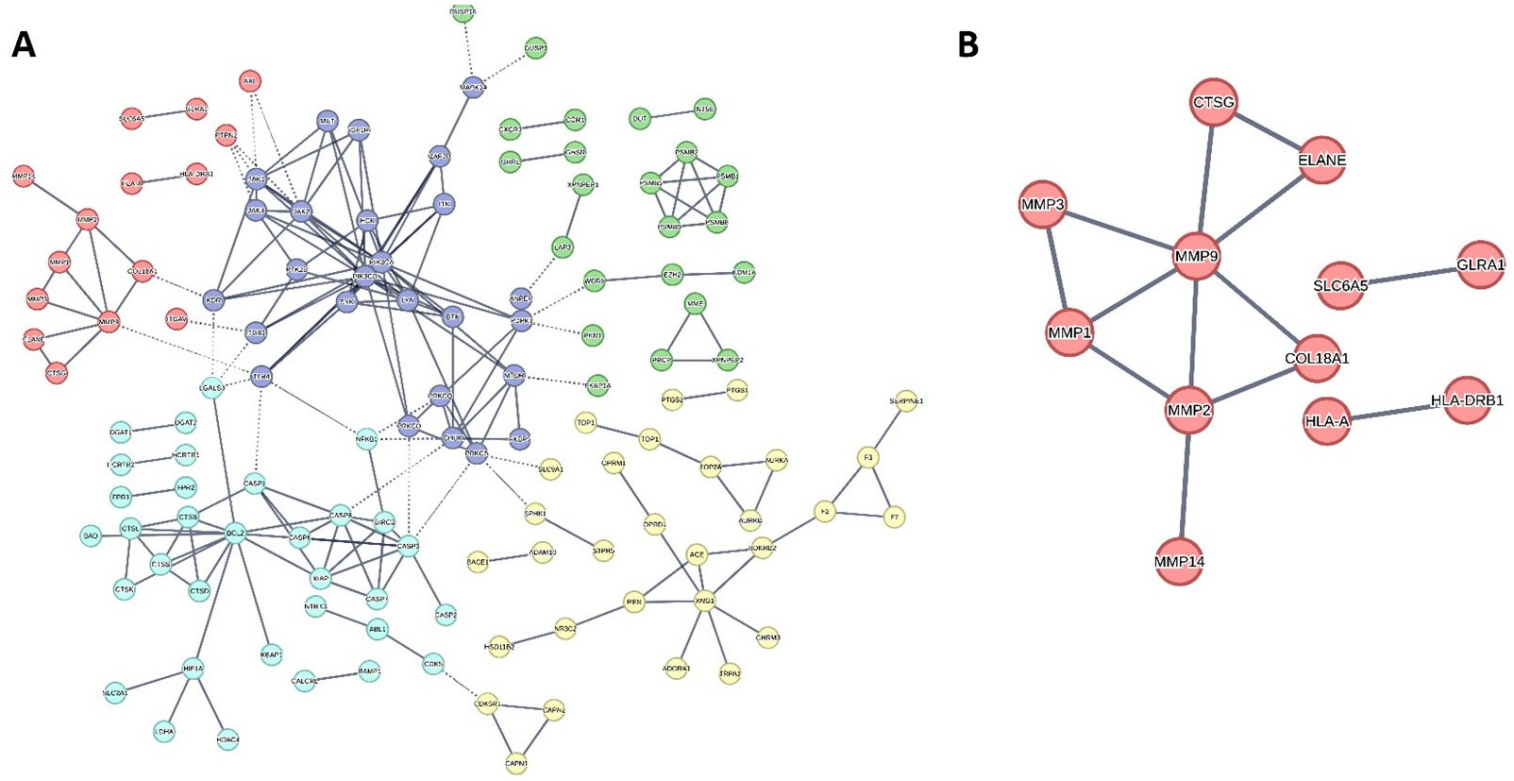
**(A)** Wound healing-relevant network categorized into 5 different subnetworks. These subnetworks represent the first 5 key pathways involved in the wound-healing process. Red nodes cluster represents the remodeling extracellular matrix pathway; Blue nodes cluster represents the PI3K-Akt signaling pathway; Green nodes cluster represents the proteasome signaling pathway; Cyan nodes cluster represents the apoptosis signaling pathway; Yellow nodes cluster represents the TNF signaling pathway. (**B**) the cluster of remodeling extracellular matrix proteins.

Furthermore, our analysis has organized the proteins within the existing network based on their roles in diverse signaling pathways linked to the wound healing process. This classification was informed by utilizing the KEGG database [available at https://www.genome.jp/kegg/pathway.html] for conducting protein enrichment analysis. The proteins depicted in the (PPI) network (as illustrated in Fig 8) were grouped into five categories, each representing a critical wound healing-related pathway. These include: (i) remodeling extracellular matrix; (ii) PI3K-Akt; (iii) proteasome; (iv) apoptosis; (v) TNF pathway (Fig 9).

In summary, the constructed PPI network for wound healing offers a concise overview of the interacting proteins and their respective signaling pathways. This highlights pivotal proteins that play a significant role in disease progression and are potential candidates for therapeutic intervention.

#### 3.3.2. Predicting target proteins for the LC-MS annotated compounds

**Fig 10:**
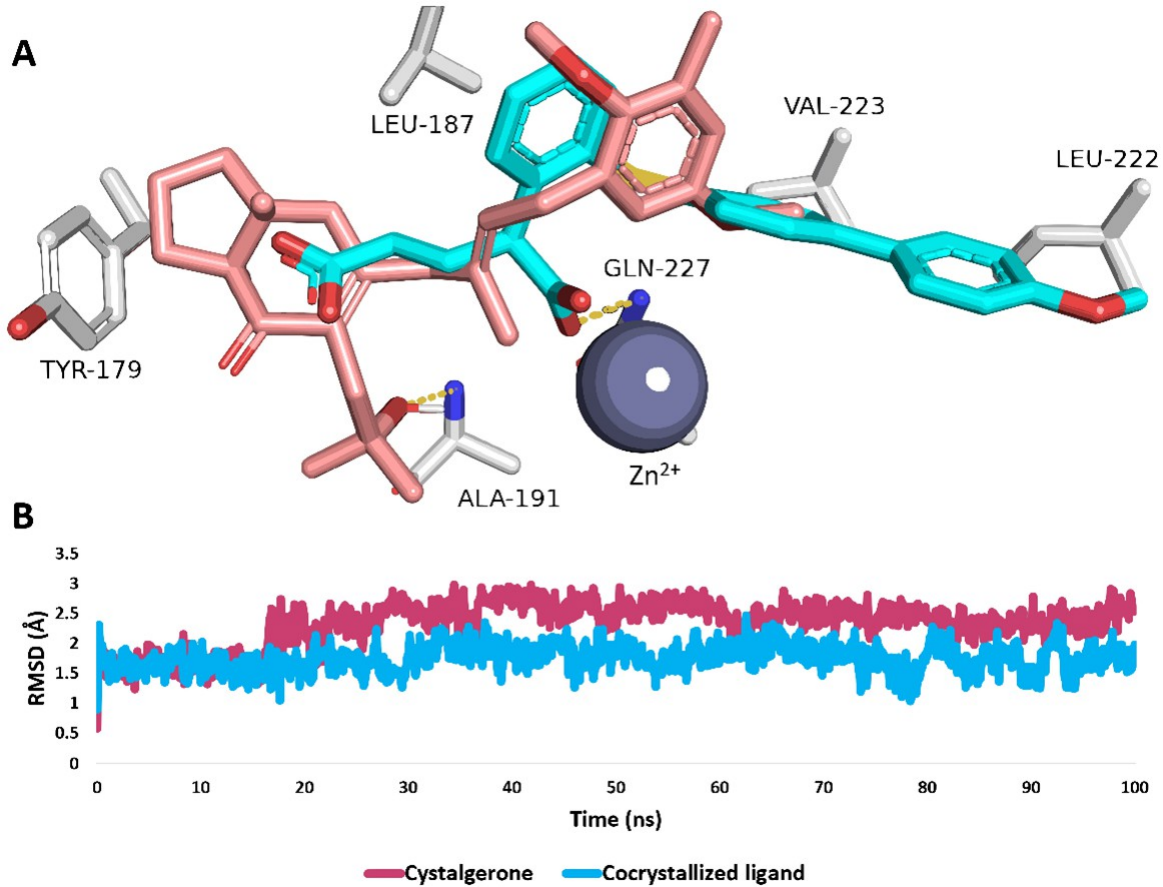
**(A)** Binding modes of cystalgerone (brick red-colored structure) in alignment with the co-crystallized inhibitors (cyan-colored structures) inside the active site of MMP9 (PDB ID: 6ESM). **(B)** RMSD profiles of both cystalgerone and the co-crystallized inhibitor inside the active site of MMP9 over the course of 100 ns-long MD simulation.

For selecting potential targets for compounds 1–3, we adopted a threshold Fit Score of 7. The analysis predicted MMP1-3 and MMP9 as likely targets for cystalgerone. Notably, MMPs are central proteins in the wound-healing process and are involved in remodeling the extracellular matrix pathway (Fig 10). This suggests that cystalgerone may be responsible for the wound healing-promoting effect of *Cystoseira* extract.

Cystalgerone underwent further analysis through molecular docking and molecular dynamics (MD) simulation experiments, enhancing the initial findings from our pharmacophore-based virtual screening. The criteria for identifying proteins as targets for cystalgerone included docking scores with the compound lower than -7 kcal/mol and absolute binding free energies (ΔG_binding_) also below -7 kcal/mol. Meeting these conditions, all the predicted proteins were then subjected to an extended refinement process involving 100 nsec-long MD simulations. Accordingly, only MMP9 that met these criteria with a docking score of -8.93 kcal/mol and ΔG_binding_ or -8.48 kcal/mol, and hence the cystalgerone-MMP9 interaction was further analyzed by MD simulation.

#### 3.3.4. Analysis of the modes of action of cystalgerone

The alignment between cystalgerone and the co-crystallized ligand within the active site of the enzyme was observed to be comparable, enabling them to form stable bindings. This stability was evidenced by relatively minor fluctuations, with average root mean square deviations (RMSDs) of 2.53Å and 1.72Å respectively, as demonstrated in 100 nanoseconds of MD simulations (Fig 10A and 10B). Both cystalgerone and the co-crystallized ligand exhibited identical hydrophobic interactions with amino acids LEU-187, and VAL-223. However, their hydrogen bonding differed: Cystalgerone formed a single hydrogen bond with the main chain of ALA-191, whereas the co-crystallized ligand established a H-bond with GLN-227 through its carboxylate moiety, which was also able to coordinate with the Zn^2+^.

**Fig 11:**
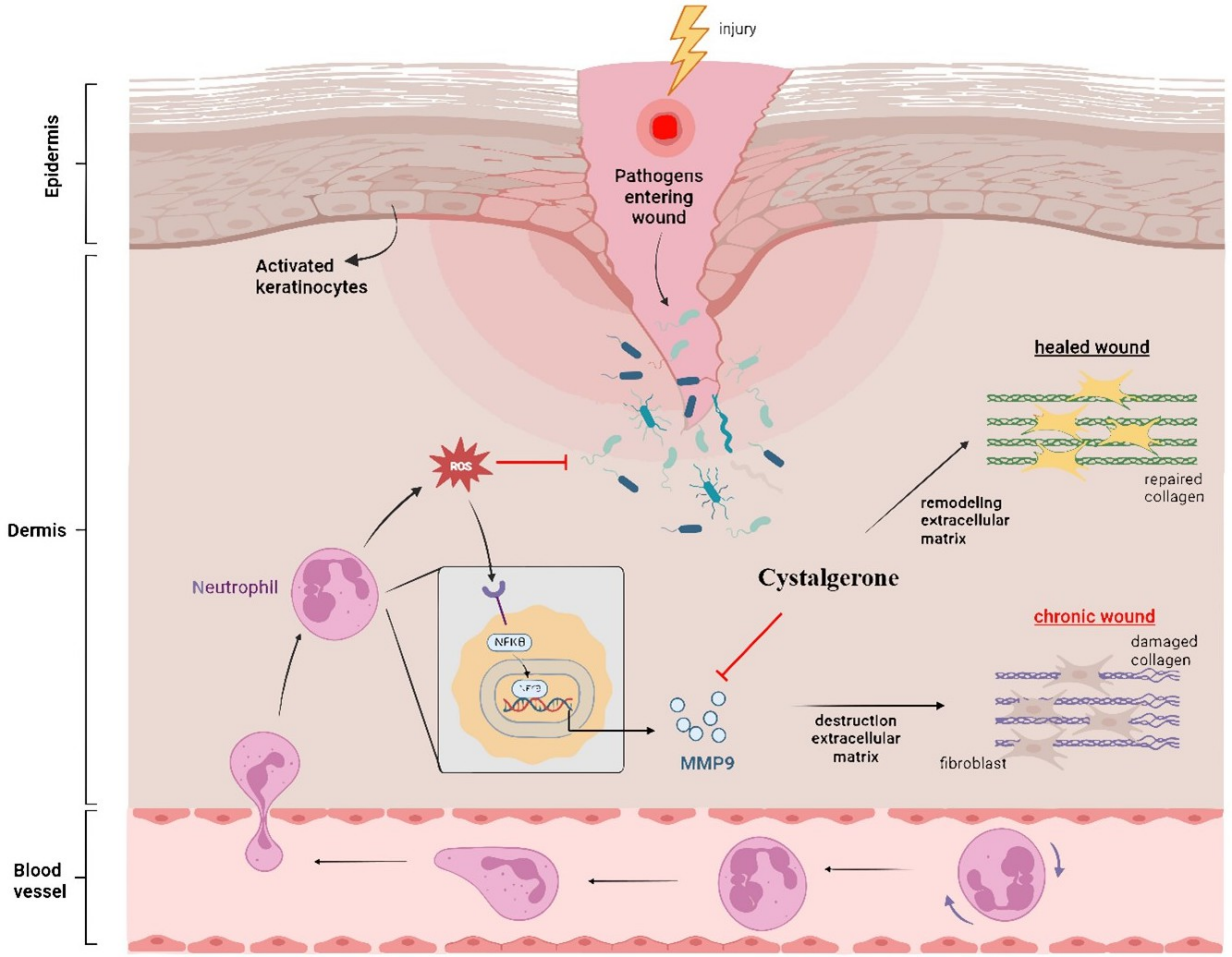
The probable role of cystalgerone in diabetic wound healing process.

An essential part of the wound-healing process is the TNF signaling pathway. Tumor necrosis factor-alpha (TNF-α), belonging to the TNF family, is a significant pro-inflammatory cytokine synthesized by macrophages in the acute inflammation phase. This presence of cytokine has also been confirmed in patients with infected wounds and ulcers, as reported by Dhamodharan *et al*. (2019) [50]. Within this signaling pathway, the inflammatory route involves the activation of NF-κB signaling, which plays a key role in the activation of MMP9 during the process of diabetic ulceration (Fig 11).

Matrix metalloproteinases (MMPs), a group of zinc-reliant endopeptidases, play a crucial role in both normal and abnormal tissue remodeling processes. These MMPs are instrumental in different stages of wound healing, effectively breaking down numerous extracellular matrix (ECM) protein components, as noted by Nagase *et al*. (2006) [51] and Yabluchanskiy *et al*. (2013) [52]. Typically, MMPs are categorized into various types, including collagenases (examples being MMP1, MMP8, MMP13), matrilysins (like MMP7), stromelysins (such as MMP3, MMP10, and MMP11), gelatinases (notably MMP2 and MMP9), and membrane type metalloproteinases (for instance, MMP14), as identified in studies by Laronha and Caldeira (2020) [53], and Rohani and Parks (2015) [54]. During the wound healing process, elevated levels of MMPs and their protease activities lead to protein breakdown, alteration of granulation tissues, modulation of angiogenesis, and regulation of growth factor activities, as discussed in research by Chang and Nguyen (2021) [55], Park *et al*. (2014) [56], and Tardáguila-García *et al*. (2019) [57].

MMP9 is composed of a catalytic domain that includes two zinc ions. This enzyme is pivotal in the breakdown of the extracellular matrix (ECM), crucial for both normal physiological functions and pathophysiological conditions involving tissue remodeling, as described by Yabluchanskiy *et al*. (2013) [52]. Additionally, MMP9 is implicated in various inflammatory conditions, including diabetes, arthritis, and cancer, as highlighted by Halade *et al*. (2013) [58]. In the context of tissue regeneration, an elevated level of MMP9 significantly contributes to delayed wound healing (Nguyen *et al*. 2018) [59] demonstrated the presence of active MMP9 in higher concentrations in severely infected wounds, as determined through affinity resin and proteomic analysis. This evidence underscores the potential of targeting MMP9 as a therapeutic approach in wound healing. In our study, we explore *Cystoseira* extract including its main metabolite cystalgerone as a possible MMP9 inhibitor, with this hypothesis backed by network pharmacology analysis.

## 4. Discussion

Wound healing is a complex process of repairing tissue layers in injured tissue as near as possible to its main architecture [4]. Surveys reported that the process of wound repair follows three consecutive phases: inflammatory owing to pro-inflammatory-mediators and immunosuppression, a proliferative phase *via* the proliferation of fibroblasts, collagen fibers, angiogenesis as well as a remodeling phase involving regeneration of injured tissues [4]. Therefore, for an effective treatment, a drug that can promote wound healing, has the potential to contribute, especially in immunocompromised patients, is natural, available, inexpensive, and has few side effects is required. There is a need to find natural medicines with benefits [60].

The LC-HR-ESI identification results revealed the prevalence of fatty acids, which were encouraging to perform the recent work because it has been documented that they have anti-inflammatory, anti-oxidant, immunomodulatory, and wound healing properties [61]. In addition, they have long been topically used in animal models for their pain relief and for their antimicrobial effects [62]. The gross evaluation results showed that the topical application of *Cystoseira* extract on the excision wounds in the immunosuppressed rats, formerly injected with BMMSCs, gave a significant reduction (p < 0.001) in the wound area compared to the control group. Nearly comparable results were obtained in the groups individually treated with the extract or the BMMSCs, with extra perfect results obtained from their combination where the wound closure rate was enhanced giving a sign of keratinocyte differentiation, angiogenesis, re-epithelialization, and fibroblast proliferation [5].

Multiple growth factors and intricate cell interactions are required for wound repair, with *TGF-β* and *IL-10* playing a critical role in all stages of the healing process. TGF-β and IL-10 recruit and activate inflammatory cells, including neutrophils and macrophages, during the hemostasis and inflammation phase. In contrast, multiple cellular responses, including re-epithelialization, angiogenesis, granulation tissue development, and extracellular matrix deposition, are created during the proliferative phase. Furthermore, fibroblasts are stimulated to proliferate and differentiate into myofibroblasts, which are involved in the wound closure step of the remodeling phase. Generally, the non-healing wounds frequently worsen a failure of *TGF-β* / *IL-10* warning, and some reports state that *TGF-β* and *IL-10* exert an inhibitory effect on the expression of collagenases as they impair collagen synthesis and extracellular matrix regeneration [1]. This is in agreement with our findings, which showed that in comparison to the wound tissues that were not treated, BMMSCs and *Cystoseira* extract optimally increased the expression of *TGF-β* and *IL-10*.

Conversely, the pro-inflammatory cytokines (TNF-α and IL-1β) are expressed to draw neutrophils and lessen impurities at the wound site; they have been identified as dynamic inducers of the synthesis of metalloproteinases (MMPs) [4]. Extracellular matrices (ECM) that have been damaged are broken down and expelled by MMPs throughout the healing process; however, if the inflammatory phase is prolonged, further tissue damage and worsening of the condition occur. Macrophages emit TNF-α, which combines with IL-1β to prevent the synthesis of collagen and the growth of fibroblasts. As a result, *NF-κB* and *INF-ϒ* are boosted by *TNF-α*, which encourages gene expression of a variety of pro-inflammatory cytokines involving *TNF-α* itself and other MMPs [4]. Thus, it is possible to reduce chronic inflammation and improve wound healing by inhibiting the production of inflammatory cytokines (TNF-α and IL-1β) (S1 Fig).

Mesenchymal stem cells produced from bone marrow (BMMSCs) exhibit multipotency, self-renewable cells existing in all post-natal organs. Despite the role of BMMSCs in reducing skin lesions, two possibilities may currently account for the explanation of the therapeutic effects of BMMSCs, which have been the subject of debate among scientists. Growth factors, cytokines, and certain proteins are examples of bioactive soluble substances that can promote the differentiation of BMMSCs into dermal and epidermal cells [6].

According to certain research, injected BMMSCs regularly move to skin injury sites and trigger the release of leukocytes, macrophages, neutrophils, and lymphocytes. Additionally, it has been shown that the secretome of BMMSCs can alter macrophage responses and polarize them to change from pro-inflammatory to anti-inflammatory during skin wound healing, which is an essential stage [11]. Also, they have been reported to inhibit the expression of pro-inflammatory cytokines, lower apoptosis, and weaken the NF-KB signal transduction pathway. Furthermore, BMMSC usage promotes ECM deposition and proliferation in addition to fibroblast migration and survival [6].

Moreover, BMMSCs have strong angiogenesis-stimulating and immunomodulatory properties [12]. Following an injury, the BMMSCs stimulate the production and growth of immunosuppressive M2 macrophages and T-regs in response to inflammatory cytokines. This reduces the negative immune response and subsequent inflammation in addition to controlling the activity of immune cells involved in tissue repair processes [63]. With the production of a large number of immunomodulatory molecules (such as *TGF-β*, NO, *IL-10*, IL-6, *IL-1β*, PGE2, *TNF-α*, and VEGF) in response to injury, BMMSCs control the immune response and vasculogenesis to improve the process of healing of damaged tissues.

Finally, the constructed PPI network for wound healing offers a concise overview of the interacting proteins and their respective signaling pathways. This highlights pivotal proteins that play a significant role in disease progression and are potential candidates for therapeutic intervention. Cystalgerone underwent further analysis through molecular docking and molecular dynamics (MD) simulation experiments, enhancing the initial findings from our pharmacophore-based virtual screening. This suggests that cystalgerone may be responsible for the wound healing-promoting effect of *Cystoseira* extract.

Collectively, when BMMSCs and *Cystoseira* extract are combined and applied on the wounded areas in immunosuppressed rats can be a promising step in the era of wounds with delayed healing, which occurs extensively in patients with diabetes, severe microbial infection, steroidal therapy, organ transplantation, or skin grafting. in the broadest context possible. Future research directions may also be highlighted.

## 5. Conclusion

In immunosuppressed patients, mesenchymal stem cells derived from bone marrow exhibit significant promise in facilitating wound healing, tissue renewal, and neovascularization. Clinical approaches have revealed a tremendous promise for BMMSCs therapy in the management of chronic and untreatable wounds. Combining BMMSCs with *Cystoseira* sp. extract to reap the benefits of their anti-inflammatory, immunomodulatory, and reparative effects present a significant challenge for modern regenerative treatment regimens, particularly in situations of immunocompromised patients. They can amplify the downregulating effect on *Cox-1*, *Cox-2*, *IL-1β*, *TNF-α*, *INF-ϒ*, and *NF-KB*, and support the upregulating effect on *TGF-β* and *IL-10*. From a constructed PPI network for wound healing and further analysis through molecular docking and molecular dynamics (MD) simulation experiments suggest that cystalgerone may be responsible for the wound healing-promoting effect of *Cystoseira* extract. Nonetheless, further investigation is needed to determine and categorize the best sources of stem cells as well as the most effective methods of delivering cells to cure wounds.

## Supporting information

S1 Fig Predicted cross talk between inflammatory and immune cells in wounded/immunosuppressed rats, dashed lines express delayed activation of pro-inflammatory cytokines and immunity markers. (TIF)

S1 Table Primers used for real-time PCR. (PDF)

S2 Table Target relevant to wound healing process. (PDF)

S3 Table Dereplicated compounds from *Cystoseira* algae. (PDF)

S1 File (ZIP)
