# Supplementary figures and images for: Wound healing potential of Cystoseira/mesenchymal stem cells in immunosuppressed rats supported by overwhelming immuno-inflammatory crosstalk
Source: PLoS One. 2024 Apr 4;19(4):e0300543. doi: 10.1371/journal.pone.0300543 (PMC10994362; doi:10.1371/journal.pone.0300543)

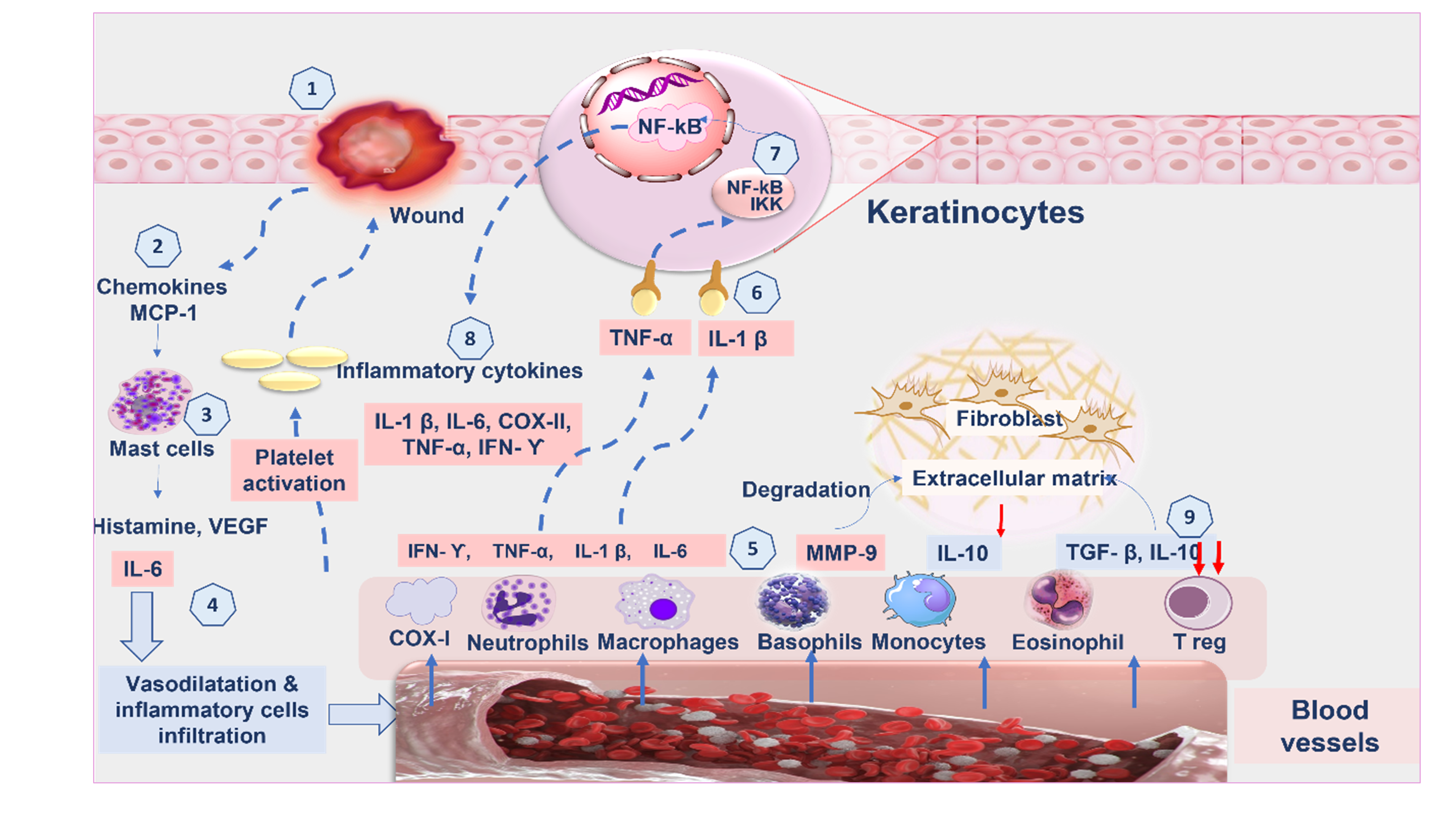

Supplement: S1 Fig — (TIF) [file pone.0300543.s001.tif]
