# Supplementary material for: Wound healing potential of Cystoseira/mesenchymal stem cells in immunosuppressed rats supported by overwhelming immuno-inflammatory crosstalk
Source: PLoS One. 2024 Apr 4;19(4):e0300543. doi: 10.1371/journal.pone.0300543 (PMC10994362; doi:10.1371/journal.pone.0300543)
Supplement: S1 Table — (PDF) [file pone.0300543.s002.pdf]

S1 Table: Primers used for real-time PCR

Name of gene	Accession number		Primer sequence
IL-1β	*NM_031512.2*	forward	5'-GTGATGAAAGACGGCACACC-3'
		reverse	5'-TCCTGGGGAAGGCATTAGGA-3'
GAPDH	*NM_017008.4*	forward	5'-CTCTCTGCTCCTCCCTGTTC-3'
		reverse	5'-CGACATACTCAGCACCAGCA-3'
TGF-β	*NM_021578.2*	forward	5'-GCTGAACCAAGGAGACGGAA-3'
		reverse	5'-GAAGTTGGCATGGTAGCCCT-3'
TNF-α	*NM_012675.3*	forward	5'-CCTCTCTGCCATCAAGAGCC-3'
		reverse	5'-GGCTGGGTAGAGAACGGATG-3'
IL-10	*NM_012854.2*	forward	5'-GCTGGAGTGAAGACCAGCAA-3'
		reverse	5'-TTCTTCACCTGCTCCACTGC-3'
NF-κB	*NM_001276711.2*	forward	5'-CAGCAGATGGCCCATACCTT-3'
		reverse	5'-CTGTCATCCGTGCTTCCAGT-3'
INF-ϒ	*NM_138880.3*	forward	5'-TGAAAGACAACCAGGCCATCA-3'
		reverse	5'-CACCGACTCCTTTTCCGCT-3'
Cox-1	*NM_017043.4*	forward	5'-TTCCGTGTGCCAGATTACCC-3'
		reverse	5'-GTTGCCAACAAGGAGCCAAG-3'
Cox-2	*NM_017232.4*	forward	5'-TTCGGGAGCACAACAGAGTG-3'
		reverse	5'-CAGCGGATGCCAGTGATAGA-3'
