# Supplementary material for: Wound healing potential of Cystoseira/mesenchymal stem cells in immunosuppressed rats supported by overwhelming immuno-inflammatory crosstalk
Source: PLoS One. 2024 Apr 4;19(4):e0300543. doi: 10.1371/journal.pone.0300543 (PMC10994362; doi:10.1371/journal.pone.0300543)
Supplement: S2 Table — (PDF) [file pone.0300543.s003.pdf]

S2 Table: Target relevant to the wound healing process

Super PRED	SWISS Target	SEA Target
CTSD	DGAT1	GHRL
NFKB1	REN	VEGFA
GPR55	MMP3	HDAC4
CTSB	MMP9	GHSR
TRIM24	MMP8	PPIA
LNPEP	MMP1	ACKR3
NTRK3	CAPN2	BIRC3
CTSL	ACE	
FKBP1A	MMP2	
MMP2	GHSR	
HDAC10	F2	
KEAP1	CASP3	
CDK5	P2RY12	
DUT	ZAP70	
CNR2	CASP8	
TMPRSS6	PSMB8	
PTGS1	PSMB5	
BLM	JAK3	
KDM1A	JAK1	
CYP19A1	JAK2	
GLRA1	OPRM1	
PTPN2	FKBP1A	
CACNA1B	TSPO	
HRH3	CTSK	
ACHE	CASP2	
MMP8	HLA-DRB1	
GABRA1	PDE10A	
KLF5	PPIA	
HDAC4	CTSB	
HIF1A	BACE1	
ABL1	PDE5A	
PIK3CD	SOAT1	
TLR8	PAM	
ATG4B	CASP1	
CSNK2B	MMEL1	
PKN1	ABCB1	
PPIA	CASP7	
TDP1	XIAP	
MMP9	CDK5R1	
FPR1	MGAT2	
CLK4	BTK	
ITK	DGAT2	
PSMB1	MAPK14	
LDHA	PDE4B	
S1PR5	PDE7A	
NR1I2	CXCR3	
MAOA	HLA-A	

ADAM10	CTSL	
TOP2A	CTSD	
LGALS3	AURKB	
HDAC11	PRKCD	
CHUK	PRKCQ	
STING1	AURKA	
ANPEP	CALCRL	
ABCC1	SYK	
EZH2	CETP	
DUSP3	ANPEP	
PRSS1	NMBR	
SLC9A1	CASP6	
GUSB	BCL2	
GRIN1	PLA2G7	
CYSLTR2	TYRO3	
KLK5	CTSS	
PSMB9	LAP3	
BCHE	ELANE	
LDHB	PIK3CA	
MMP12	OXTR	
TRPA1	GNRHR	
PIN1	OPRD1	
SLC6A5	F3	
CAPN1	BDKRB2	
FPR2	CHRM3	
FKBP5	MTOR	
SLC2A1	PSMB2	
SLC40A1	PSMB1	
CTSG	KNG1	
ADORA2B	PTK2B	
GSTP1	MAPK10	
HTR7	XPNPEP1	
GHSR	XPNPEP2	
PHF8	IGF1R	
NT5E	CFD	
MME	ITK	
ITGB1	CTSG	
NR3C2	CTRB1	
PSMB2	HCRTR2	
TYRO3	HCRTR1	
SPHK1	PTGS2	
SERPINE1	RORC	
WDR5	PFKFB3	
C5AR1	HCK	
DCUN1D1	MET	
FCGRT	LYN	
GPBAR1	MAP4K2	
FFAR2	ITGAV	
NTSR2	EZH2	
AVPR1B	KDR	

KLK7<br>ACE<br>ZAP70<br>TLR4<br>TOP1<br>AXL<br>SCD<br>ADORA1<br>CCR1<br>TACR2<br>PRCP<br>HSD17B10<br>SCN2A<br>PRKCB<br>HSD11B2<br>PDPK1<br>MMP14	PTGER1<br>BRD9<br>BAD<br>CDK5<br>F7<br>RAMP1	
