# Supplementary material for: Wound healing potential of Cystoseira/mesenchymal stem cells in immunosuppressed rats supported by overwhelming immuno-inflammatory crosstalk
Source: PLoS One. 2024 Apr 4;19(4):e0300543. doi: 10.1371/journal.pone.0300543 (PMC10994362; doi:10.1371/journal.pone.0300543)
Supplement: S3 Table — (PDF) [file pone.0300543.s004.pdf]

S3 Table: Dereplicated compounds from *Cystoseira* algae

No.	RT (min)	Ionization (ESI+/ESI-)	Compound Name	Molecular Formula	Molecular Weight	Observed (*m/z*)	References
**1**	6.6017	$[M + H]^+$	1,6-Octadien-3-ol, 3,7-dimethyl-, 2-aminobenzoate	$C_{17}H_{23}NO_2$	273.1728	274.1727	[1]
**2**	7.6005	$[M + H]^+$	Mannitol	$C_6H_{14}O_6$	182.0790	183.0791	[1]
**3**	9.5853	$[M + H]^+$	Hexadeca, -4,7,10,13-tetraenoic	$C_{16}H_{26}O_2$	250.1932	251.1931	[1]
**4**	10.0323	$[M + H]^+$	2,3-Dihydroxypropanoic acid; (R)-form, 2-O-α-D-mannopyranoside	$C_9H_{16}O_9$	268.0794	269.0795	[2]
**5**	10.2939	$[M + H]^+$	1,4-Dihydroxy-2,7(19),10,14-phytatetraen-13-one; (2E,4R,10E)-form, 4-ketone	$C_{20}H_{30}O_3$	318.2194	319.2190	[3]
**6**	10.6000	$[M + H]^+$	6,10,14-Trimethyl-5-pentadecene-2,12-dione; (E)-form	$C_{18}H_{32}O_2$	280.2402	281.2401	[4]
**7**	11.1829	$[M + H]^+$	2,6,10,14-Phytatetraene-1,13-diol; (2E,6E,10E,13R)-form, 13-ketone	$C_{20}H_{32}O_2$	304.2402	305.2404	[5]
**8**	11.6459	$[M + H]^+$	Zosterdiol A; 1',4'-Di-de-Me, 1',4'-quinone, 5-Ac	$C_{29}H_{40}O_6$	484.2824	485.2826	[6]
**9**	12.2231	$[M + H]^+$	α-tocopherol	$C_{29}H_{50}O_2$	430.3810	431.3813	[1]
**10**	12.9077	$[M + H]^+$	Cystalgerone; O1'-De-Me	$C_{28}H_{40}O_4$	440.2926	441.2928	[7]
**11**	12.9648	$[M + H]^+$	Eicosanoic acid; amide	$C_{20}H_{41}NO$	311.3188	312.3186	[8]
**12**	13.2150	$[M + H]^+$	2,13-Octadecadien-1-ol	$C_{18}H_{34}O$	266.2609	267.2609	[9]
**13**	14.1155	$[M + H]^+$	1-(2-Hydroxy-5-methoxy-3-methylphenyl)-3,7,11,15-tetramethyl-2,6,10,14-hexadecatetraene-5,12-diol; (2E,5R,6E,10E,12R)-form, 12-ketone	$C_{28}H_{40}O_4$	440.2926	441.2927	[5]
**14**	15.0007	$[M + H]^+$	Cystalgerone	$C_{29}H_{42}O_4$	454.3083	455.3085	[10]
**15**	15.3373	$[M + H]^+$	3,10(18)-Pachydictyadiene-6,14,15-triol; (1α,5β,6β,11R,14S)-form, 6-Ac	$C_{22}H_{36}O_4$	364.2613	365.2615	[11]

RT: Retention time; min: Minute
